# Measurement of Light Absorbing Aerosols with Folded-Jamin Photothermal Interferometry

**DOI:** 10.3390/s20092615

**Published:** 2020-05-04

**Authors:** Jeonghoon Lee, Hans Moosmüller

**Affiliations:** 1 School of Mechanical Engineering, Korea University of Technology and Education, Byeongcheon-myeon, Cheonan 31253, Korea; 2 Laboratory for Aerosol Science, Spectroscopy, and Optics, Desert Research Institute, Nevada System of Higher Education, Reno, NV 89512, USA; Hans.Moosmuller@dri.edu

**Keywords:** photothermal interferometry, light absorption, aerosols, black carbon

## Abstract

In this study, a photothermal interferometer was developed, based on a folded-Jamin polarization instrument with refractive-index sensitive configuration, in order to characterize light-absorbing aerosols. The feasibility of our interferometric technique was demonstrated by performing photothermal spectroscopy characterizing spark-generated black carbon particles with atmospherically relevant concentrations and atmospheric aerosols in a metropolitan area. The sensitivity of this interferometric system for both laboratory-generated aerosols and atmospheric aerosols was ~ 1 (μg/m^3^)/μV, which is sufficient for the monitoring of black carbon aerosol in urban areas.

## 1. Introduction

The light absorption of atmospheric aerosols, such as black carbon (BC), is important because it affects climate forcing through the direct and semi-direct interaction with solar radiation [1,2]. Thus, the quantification of BC in the atmosphere becomes critical for the estimation of the radiative forcing of the atmosphere [3]. However, quantifying the light absorption of atmospheric BC is still a challenging task. Currently, filter-based techniques using light attenuation are widely used as a tool for the characterization of BC due to their easy field deployment as well as their stable performance in the laboratory [4,5,6]. Another method used to characterize BC without filter deposition, laser-induced-incandescent (LII) type instruments are also capable of characterizing coatings on BC [7,8]. The photo-acoustic technique is a very direct, in situ measurement of the aerosol absorption coefficient at the wavelengths of the light source employed [7]. Here, we describe the utilization of photothermal interferometry (PTI) based on a polarized folded-Jamin interferometer configuration for absorption spectroscopy of airborne particles without deposition of aerosols on a filter.

Interferometric techniques associated with PTI have been implemented for about three decades since the 1980s when Mach-Zehnder type interferometers were introduced to measure light absorption of not only aerosols but also chemical species [9,10,11]. A Jamin interferometer was successfully implemented for the detection of gas-phase ammonia [12]. The development of a folded-Jamin style interferometer [13] had a strong influence on the development of a modified folded-Jamin interferometer [14,15]. The modified folded-Jamin interferometer uses an approach to split the retroreflector widthwise into two parts and to connect these two halves together through a PZT [15]. Recently, an image-based PTI was attempted using a Mach-Zehnder configuration [16], and a laboratory-based four-channel detection scheme utilizing a folded-Jamin interferometer has been tried for better sensitivity [17]. A fiber-optics-based PTI was recently developed for the detection of gas [18]. 

As previously summarized in review papers [7,8], local heating of aerosols by light absorption induces a change in the refractive index of the medium (e.g., air) surrounding the absorbing particles. This refractive index change can be detected with an interferometer employing a stable single-frequency laser, such as a He-Ne laser.

To the best of our knowledge, this is the first time that polarization interferometry is applied to the measurement of light-absorbing aerosols. Thereby, we demonstrate the use of photothermal interferometry for the measurement of light-absorbing aerosols and its current limitations. Data from the polarization-based PTI were collected for BC aerosol generated with a spark discharge. The sensitivity of this PTI was estimated for 60-nm diameter, spark-generated BC particles. PTI signals were compared with equivalent BC (eBC) mass concentrations measured with a filter-based instrument and the PTI sensitivity was estimated from the PTI signal and the eBC concentrations measured at an urban field site. This paper demonstrates the facilitation of photothermal interferometry as a potential instrument to measure light-absorbing aerosols. We close with a realizable suggestion about improvements on sensitivity and limitation, for further development.

## 2. Methods and Apparatus

### 2.1. Experimental Setup of the PTI System

This study focuses on the development of a polarization-based photothermal interferometer system and its application for the characterization of laboratory-generated BC and ambient BC through the comparison of its data with those of commercially available instruments. Our technique is based upon the interferometric measurement of optical path variations in a folded-Jamin configuration. Relaxation of the photothermally excited mode of light-absorbing aerosols results in local heating of the surrounding medium, generating a local variation in the density and refractive index of the medium. Detection of the refractive index change involves the integration of the signal over the measurement volume. The temperature variation concomitantly creates the change in the refractive index in the surrounding air along an optical path. A phase delay or optical pathlength change results and the aerosol light absorption can be characterized by the optical pathlength change [13].

**Figure 1:**
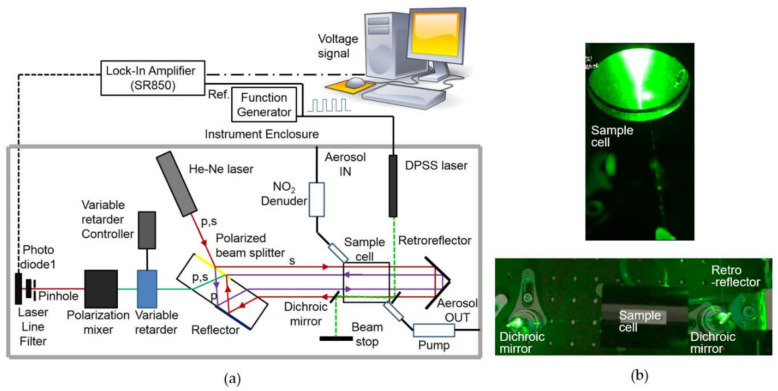
(**a**) Schematic of the photothermal interferometry (PTI) system used in this study. (**b**) Side view and top view of the sample cell.

Basically, our interferometer consists of a He-Ne laser, a polarized beamsplitter plate, a retroreflector, a variable retarder, a polarization mixer (polarized beamsplitter), and two photodiodes [13]. The interferometer setup and principle of operation were described previously [13]. A diode-pumped-solid-state (DPSS) laser (MGL-H-532/500~1200mW, CNI Laser, China) was added to the setup as a pump laser to heat light-absorbing particles in the measurement volume. A sample cell, two dichroic mirrors, a lock-in amplifier, and a function generator were added to the interferometer to complete our optical setup. The entire experimental setup is schematically depicted in Figure 1. A single-frequency He-Ne laser (HRS015B, Thorlabs, USA) was used to generate an interferometer source beam. Optical isolation of the reflected beam was accomplished by a slight tilting of optical components. An aperture was placed in front of the photodiode detector to reduce the influence of straylight.

Observation of both laser beams confirmed that the profile of the He-Ne laser beam (probe beam) can be described by a transverse electromagnetic mode (TEM_00_) while that of the DPSS pump laser beam was nearly TEM_00_. Both pump and probe beams were aligned nearly coaxial with the sample cell and focused to beam waists (radius at half-field strength) of approximately 1 mm, defining the measurement volume of the instrument. The excitation source was a continuous-wave (CW) 532-nm wavelength DPSS laser that was power-modulated with an 83-Hz frequency TTL signal. The DPSS beam was coupled into and out of the sample cell with dichroic mirrors to maximize the overlap between the probe and pump beams in the measurement volume of the sample cell. A band-pass filter centered at a wavelength of 633 nm blocked the 532 nm wavelength pump beam from detection by the photodiode and a near-zero signal was present in the absence of the He-Ne probe beam.

A 100-mm long annular denuder (URG-2000-30x100-3CSS, URG, USA) consisting of Teflon-coated stainless steel with guaiacol [19] was installed in front of the inlet line to remove nitrogen dioxide (NO_2_) that absorbs light in most of the visible range (e.g., [20]). The sample cell was a stainless-steel cylindrical tube of 70 mm length and 45 mm inner diameter and 55 mm outer diameter with an inlet and an outlet mounted laterally onto the surface of the sample cell. The sample cell was sealed by two rubber O-rings at both sides. The volume of the sample cell was approximately 0.11 liters. Float glass optical windows were installed with AR coating. Outer surfaces of windows were cleaned with HEPA-filtered N_2_ when needed. Inner surfaces of windows were cleaned whenever it was purged with HEPA-filtered N_2_. It was used with a typical sample flowrate of 0.3 l/min.

A function generator (DG1062, Rigol, USA) provided a TTL signal modulating the power of the DPSS (i.e., pump) laser. Internal power modulation with a TTL signal, instead of using a mechanical chopper, greatly reduces coherent acoustic noise. PTI signals were analyzed with a lock-in-amplifier (SR850, Stanford Research System, USA) to remove noise and improve the signal-to-noise ratio. 

The components discussed above were mounted in an inner and outer enclosure for improved environmental isolation and laser safety. The inner enclosure, a rectangular black aluminum housing, contained the specially manufactured polarizing beams splitter, the Porro-prism-type retroreflector, the customized sample cell, two dichroic mirrors, the liquid crystal variable retarder, the polarization mixer (a polarizing beam splitter) and the photodiode. Other components, including the He-Ne laser, the DPSS laser, the flow pump, and the NO_2_ denuder were surrounded by the outer enclosure for better stability and blocking of ambient acoustic noise and for constraining laser light to its inside. The instrument was installed on top of a pneumatic optical table to isolate it from ground vibration.

### 2.2. Production of Spark-Generated BC, Selection of Size, and Measurement of Number Concentration

A spark discharger (DNP 2000, PALAS GmbH, Germany) generated black carbon (BC) through high voltage sparks between graphite electrodes. Generated BC particles were further size selected and their number concentration was quantified with a differential mobility analyzer (DMA, home-made) equipped with a condensation particle counter (CPC, TSI 3775, USA). Mono-disperse BC particles with a specific size were injected into the sample cell of our PTI system and their PTI signal was measured.

### 2.3. Characterization of Ambient Equivalent Black Carbon (eBC)

Ambient PTI data were compared with eBC mass concentrations measured with a multi-angle absorption photometer (MAAP 5012, Thermo Scientific, USA) installed at the monitoring site (37.521°N, 127.124°E) in Olympic Park in Seoul, South Korea. MAAP measurements of aerosol absorption coefficients were shown to be in excellent agreement with reference measurements [21], the MAAP instrument and its operation have been described in detail [22], and more recently, its long-term (> 1 month) performance was verified through a continuous measurement [23].

### 2.4. Estimation of Effective Density and Mass Concentration of Spark-Generated BC

We assume that the *effective density* is correlated with the fractal dimension and mobility diameter as follows

(1) $$\rho_{eff}\sim d_{m}^{D_{f}-3},\;$$

where *ρ_eff_* is the effective density, *d_m_* is the mobility diameter, and *D_f_* is the fractal dimension [24]. *D_f_* has long been regarded as the fractal dimension in this equation though it is more relevant to refer to it as the mass-mobility exponent [25]. In this paper, we compared our *D_f_* to various ones obtained from different techniques such as light scattering, image-analysis, and mass-mobility measurements as an approximate way to show that our approach makes sense because our approach is novel. The fractal dimension *D_f_* was determined by curve-fitting the effective density of spark-generated BC (or PALAS soot) as a function of the mobility diameter previously measured [26]. With the resulting fractal dimension of *D_f_* = 1.89 or 1.95, the effective density was determined as a function of mobility diameter *d_m_* using Equation (1). The effective density is required to estimate the mass of BC from the measured *effective volume,* which is calculated as *πd_m_^3^/6*. The effective volume implies the entire volume of the spark-generated BC and is calculated as a function of the mobility diameter. Then, the *effective mass* is easily calculated by multiplying the effective density and the effective volume. Consequently, the effective mass concentration is obtained by simply multiplying the effective mass with the number concentration measured with the CPC. 

## 3. Results and Discussion

### 3.1. Set Up for the Quadrature Condition

**Figure 2:**
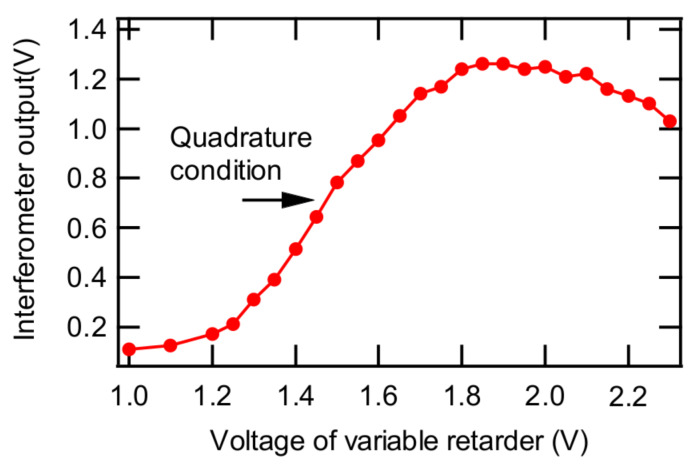
Interferometer signal measured as a function of the voltage of variable retarder.

**Figure 3:**
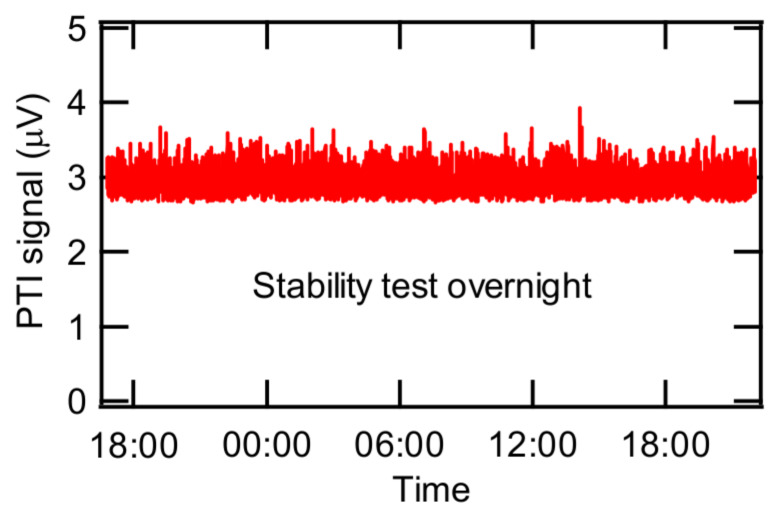
Stability test for PTI signal at a fixed voltage of the variable retarder controller.

The PTI output is most sensitive at the quadrature condition, which is obtained at the zero-crossing of the signal for an ideal (e.g., sinusoidal) curve [27]. The quadrature condition can be obtained by adjusting the control voltage of the variable retarder such that the derivative of the PTI output becomes maximum. In this study, the quadrature condition was actualized by monitoring the average value of the minimum and the maximum PTI outputs. Figure 2 shows an example of the PTI output from the photodiode as a function of the control voltage of the variable retarder. PTI output increased up to a maximum value and then decreased as the control voltage further increased. As shown in Figure 2, the PTI output at the quadrature was 0.757 V, which was too high to be detected by our lock-in amplifier. To overcome this overload issue, a neutral density filter was installed in front of the photodiode to reduce the output signal from the photodiode to less than 0.1 mV, the maximum input of our lock-in amplifier. In addition, a laser line optical filter was installed in front of the photodiode to remove any light except the interferometer laser light (633 nm, He-Ne laser). It was confirmed that the PTI signal at a fixed voltage of the variable retarder controller was stable for about 24 hours as shown in Figure 3 and that the signal was sensitive to the controller voltage.

To find an experimental noise limit, we demonstrated that the performance of PTI depends on the detectable background noise acquired by the lock-in amplifier. This noise level was obtained while both the He-Ne laser and DPSS laser were simultaneously turned on, traversing the measurement volume without any aerosol injection for ~15 h. The time constant of the lock-in amplifier was set to 10 s for this background noise experiment. The average baseline signal was determined to be 2.34 ± 0.91 μV and this determines the low detection limit of our PTI system to be ~2.7 μV or 2.9 μV Hz^−0.5^. In our current PTI system, this maximum sensitivity was attained at certain retardation resulting from the phase being adjusted to quadrature condition. This is the estimation of the total system sensitivities attainable with our PTI instrument. Theoretically, it is possible to measure absorption coefficients of less than 0.01 Mm^−1^ or 0.0015 μV, assuming that mass-absorption-cross-section of BC is 6.6 m^2^/g. However, the sensitivity of our PTI system was likely further limited by absorption on the cell windows (e.g., [9]).

### 3.2. Laser Stability

**Figure 4:**
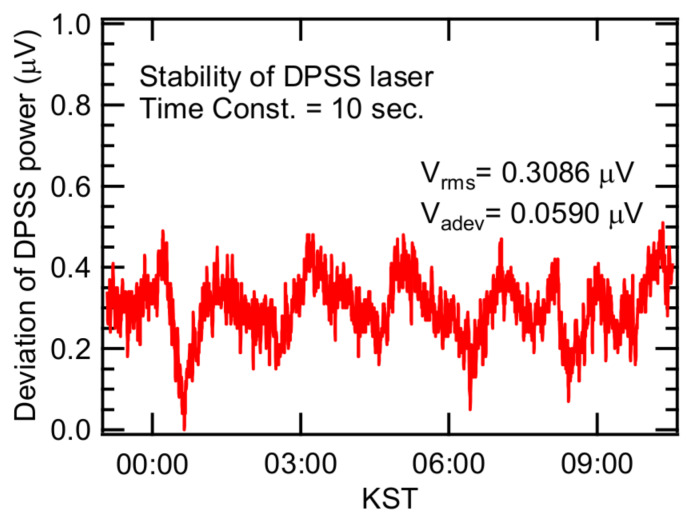
Periodic instability caused by one of the noise sources, pump laser.

Figure 4 provides ~10 h of monitoring data for the DPSS laser (pump laser), which showed intrinsic instability. Periodic drift was observed during monitoring of the signal detected at the photodiode while the DPSS laser (pump laser) was turned on and the He-Ne laser (interferometer laser) was turned off. Some of the signal detected by the photodiode seems to originate from the pump laser light that was not perfectly excluded from the photodiode, likely originating from scattering or diffraction of pump laser light by optical components. Practically, it is impossible to avoid this kind of noise signal. Thus, we call this a ‘characteristic system noise’. Optical components do not perfectly reflect and transmit the laser beam because losses due to defects exist in all optical components. Noise from such defects manifests itself throughout the system because the DPSS laser beam passes through multiple optical components including lenses and beam splitters. Thus, it is quite plausible that the periodic noise signal shown in Figure 4 originated from the unstable output of the pump laser. This effect from the DPSS laser output oscillated between 0 and 0.5 μV (Figure 4). FFT analysis of this signal resulted in a peak at a frequency of 0.145 mHz or a period of oscillation of 1 hr 52 min; this periodic oscillation is obvious in Figure 4. The analysis regarding the unstable laser source allowed us to estimate some performance parameters of our PTI system. The average signal from the instability of the DPSS laser was 0.31 ± 0.06 μV, which is present when the DPSS laser is turned on.

### 3.3. Detection of Spark-Generated BC Particles

**Figure 5:**
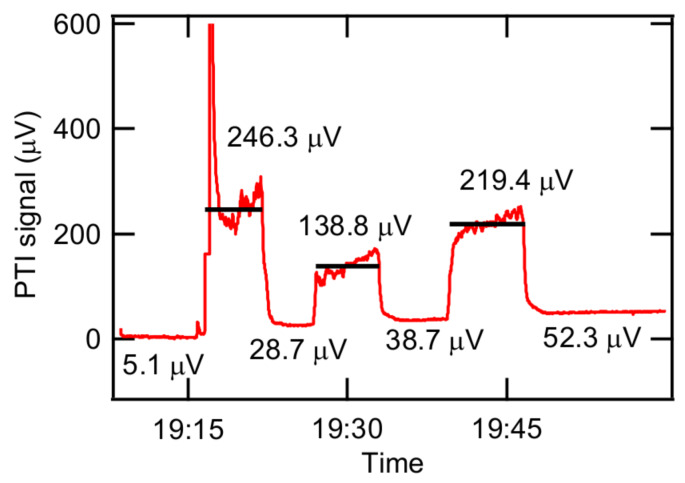
Response to spark-generated BC particles with different concentrations.

**Figure 6:**
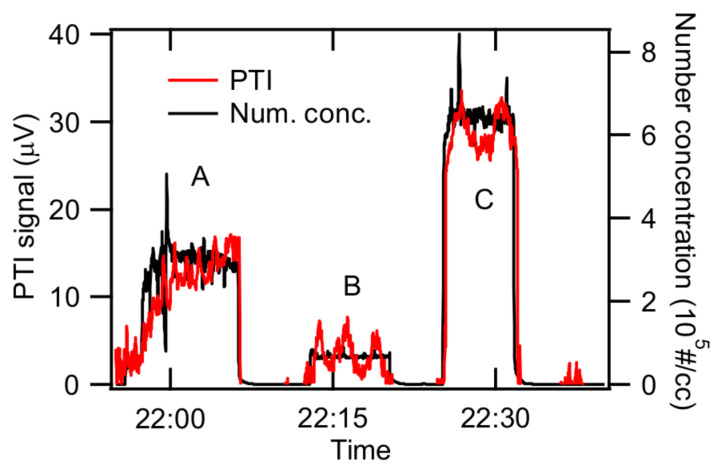
Response of PTI to known concentrations of size-selected, spark-generated BC particles.

We tested the response of our PTI system to light-absorbing particles with laboratory-generated, light-absorbing aerosols (spark-generated BC particles) with arbitrary concentration. The results are shown in Figure 5, where different concentrations of spark-generated BC particles were introduced into our sample cell three times, interspersed with a purging of the sample cell and conduit line with HEPA-filtered air. The average PTI signals during the injection of BC particles and HEPA-filtered air are shown in Figure 5 as legends. PTI signals drastically increased when BC particles were introduced into our PTI system. At the first injection, the PTI signal gradually increased and the average value was 246.3 μV. Unexpectedly, we observed an increase of 23.6 μV in the baseline. At the second and third injections, the baseline increased by 10.0 and 13.6 μV, respectively. The first injection produced the highest increase in PTI signal among three injections and the increase in the baseline was also highest at the first injection. Thus, the baseline increase is likely related to the amount of BC injected into the sample cell. We suspect that some residual BC aerosol still existed after purging and that some BC was also deposited onto the sample cell windows, both increasing the baseline signals after BC injections. This situation was improved by installing a small fan ejecting heat from the PTI system and by purging residual BC with HEPA-filtered nitrogen (N_2_) rather than with air to alleviate the accumulation of particles on the surface of the sample cell, resulting in the prevention of the baseline increase. Injecting smaller numbers of BC particles also helped to mitigate the deposition onto the surface of the sample cell. As shown in Figure 6, the baseline did not increase much (only ~0.4 μV) after installing the small fan, using HEPA-filtered N_2_, and introducing low numbers of particles. Surprisingly, as seen from Figure 6, the increase in baseline scaled with the BC concentration injected, as 0.102 μV/(μg/m^3^) with R^2^ = 0.91 assuming that the sensitivity of the current system is 1 (μg/m^3^)/ μV. Perhaps, reducing BC deposition on the surface of windows and optics may improve our system sensitivity.

### 3.4. Characterization of Spark-Generated BC

The feasibility of our PTI system was demonstrated in a calibration experiment using the size-selected BC generated by the spark-discharger. We produced BC with a mobility diameter of 60 nm. First, we injected BC particles (indicated as A in Figure 6) and then flushed with particle-free N_2_. Next, we injected lower number concentrations of BC particles (indicated as B in Figure 6) and then flushed with particle-free N_2_. Finally, we injected BC particles with the highest number concentration (indicated as C in Figure 6). As shown in Figure 6, the average PTI signal for case A was 12.3 ± 2.85 μV when the number concentration was 2.93 (±0.443) × 10^5^ #/cc. PTI signal for case B was 3.55 ± 1.67 μV when the number concentration was 6.77 (±0.43) × 10^4^ #/cc. The PTI signal for case C was 28.9 ± 1.96 μV when the number concentration was 6.44 (± 0.29) × 10^5^ #/cc. These number concentrations and PTI signals are summarized in Table 1.

The effective density of 60-nm diameter, spark-generated BC was extrapolated from the effective densities of 200-nm, 304-nm, and 500-nm diameter BC measured by [26], who reported multiple effective densities, either 0.075 g/cc or 0.082 g/cc, for 304-nm diameter BC. If the absorption cross-section and the particle mass are measured, the effective mass-absorption-cross section (MAC) can be obtained by dividing the absorption-cross section by the particle mass.

**Figure 7:**
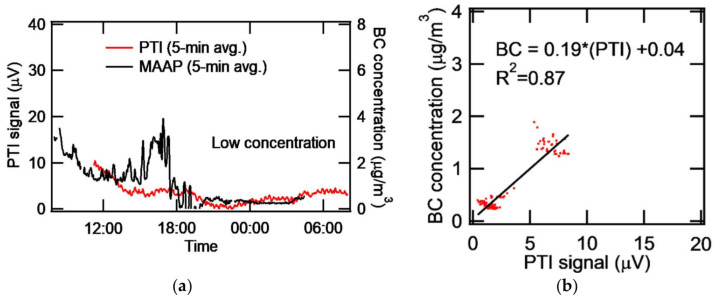
(**a**) Chi-square plot vs. fractal dimension for two different effective densities. (**b**) Extrapolation of the effective density using the values obtained from Figure 7a.

The chi-square values were calculated and plotted for various fractal dimensions. Figure 7a shows that the chi-square has the minimum at *D_f_* = 1.95 for *ρ_eff_dm=304 nm_*=0.082 g/cc and *D_f_* = 1.89 for *ρ_eff_dm=304 nm_*=0.075 g/cc. A combination of tandem differential mobility analyzer (DMA) with an electrical low-pressure impactor (ELPI) showed that the fractal dimension varied from *D_f_* = 2.15 ± 0.10 for flame-generated soot to *D_f_* = 2.30 ± 0.10 for vehicle exhaust particles [28]. A transmittance-electron-microscope (TEM) image analysis yielded a fractal dimension of 1.82 for flame generated soot [29].

An angular dependent static light scattering technique determined *D_f_* = 1.70 ± 0.10 for flame-generated soot aggregate [30]. This literature survey for the fractal dimension shows that it ranges from 1.60 to 2.16 for flame-generated soot depending on the measurement techniques and flame conditions. For spark-generated carbon, the mass-mobility fractal dimension was measured to be 2.16 using a combination of a scanning mobility particle sizer (SMPS) and a cascade epiphaniometer (CEPI) [31], and was 1.60 measured with static light scattering [32]. In the present study, the fractal dimension was estimated with curve-fitting using the existing effective densities as a function of mobility diameter. The fractal dimensions of 1.89 and 1.95 estimated in the present study lie in the range of literature values for spark-generated carbon as well as flame-generated soot, being not unreasonable in terms of morphological characteristics.

Using Equation (1) with the fractal dimension determined above and a proportional constant obtained from the curve fitting, the effective density of 60-nm diameter spark-generated BC was extrapolated and ranged from 0.403 to 0.428 g/cc as shown in Figure 7b. The mass concentration of the BC can be easily obtained as explained earlier and those values are listed in Table 1. The absorption cross-section can be estimated from the absorption efficiency of spherical particles [33]. The absorption cross-section depends on the primary particle size of aggregates, the complex refractive index, and the wavelength of the light. In the present study, PTI uses a pump laser beam with a wavelength of 532 nm. The refractive index of black carbon was reported to be 1.95 + 0.65*i* [34] which we assumed in this calculation. The primary particle size for the spark-discharged BC was reported to be 36.3 ± 13.0 nm [35]. From these values, the MAC was calculated to be 4.15 m^2^/g for the mean size (36.3 nm). The MAC ranges from 1.10 to 10.4 m^2^/g, taking into consideration the distribution of the primary particle size with standard deviation of 13.0 nm. These MAC values can be compared to the conventionally used MAC at 550 nm, 7.5 m^2^/g [36]. Though spark generated carbon is not produced by combustion, it can be regarded as BC according to Lack et al. [7], which defines it as ‘graphitic carbon with aggregate morphology’. Spark discharge produces carbon particles using two graphite rods and the observation of spark-generated carbon with a scanning electron microscope (SEM) has demonstrated that it consists of aggregates [35]. While there is no generally accepted reference material for BC, potential reference materials include spark discharged carbon [37]. For particle optics calculations, Mie theory does provide a first-order description of optical effects in nonspherical particles, and it correctly describes many small particle effects that are not intuitively obvious [33]. Therefore, we assumed that spark discharged carbon is spherical to perform simple Mie calculations of particle optics with limited information. However, in reality, the particles are fractal-like chain aggregates with a mobility diameter of 60 nm. We can imagine that the aggregates comprise two or three primary particles having individual mobility diameters of 36.3 nm. Sensitivity was estimated from the standard deviation of the PTI signal for the spark-generated BC. The sensitivity of PTI to 60-nm diameter, spark-generated BC approximately ranged from 0.87 to 1.16 (μg/m^3^)/μV. The detection limit was calculated to be 1.45 to 3.29 μg/m^3^. Those values are listed in Table 1.

### 3.5. Application to the Measurement of Ambient Aerosol

**Figure 8:**
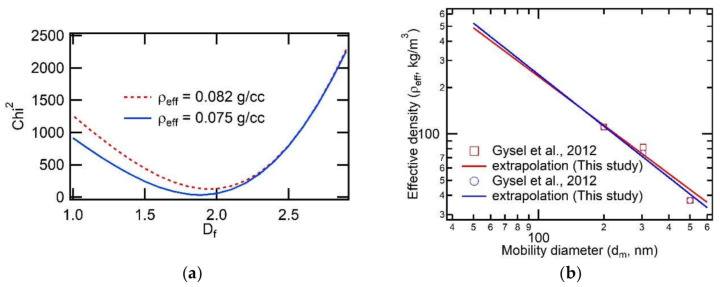
At high concentration episode (**a**) Time series of PTI and MAAP (**b**) Correlation of BC concentration and PTI signal.

**Figure 9:**
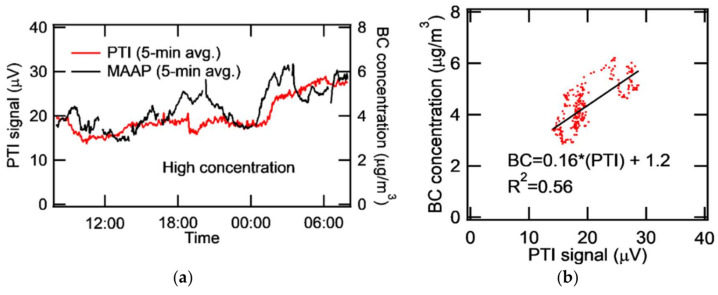
At low concentration episode (**a**) Time series of PTI and MAAP (**b**) correlation of BC concentration and PTI signal (Data from 1:00 - 6:00 p.m. were excluded for better analysis.).

Our PTI system was deployed at the monitoring site (37.521°N, 127.124°E) in Olympic Park located in a mega-city, Seoul, South Korea. The co-deployed MAAP was used to monitor and compare eBC concentration. The time constant of the lock-in-amplifier was set to 10 s and the PTI signal was collected every 10 s. Both PTI and MAAP data were box-averaged over 5 min and plotted every 5 min for better display. The diurnal variation of ambient eBC for a day of high concentration is shown in Figure 8. The PTI signal collected for 24 hours from 08:00 to 08:00 the next day (Korea Standard Time) showed a similar trend to the eBC concentration measured by the MAAP. The diurnal variation of ambient eBC for a day of low concentration is shown in Figure 9. Low noise for PTI seems attributable to improved optical alignment. The MAAP detected a higher concentration of ambient eBC around 14:00-17:00, but the PTI did not. MAAP data sometimes showed negative concentration and noisy results at continuously low concentrations of ambient eBC. When the eBC concentration measured by the MAAP was larger than that corresponding to the PTI signal, it is probable that moisture adsorption onto filter materials of the MAAP enhanced the darkness of the filter, causing the increase in light absorption. Consequently, eBC concentration could be overestimated due to adsorbed moisture. It is suspected that humid substances were entrained into the inlet system during this case. In fact, the relative humidity was 99% from 15:00 to midnight due to weak rain. The standard deviation of the PTI signal in the high concentration case was 3.68 μV and the detection limit turned out to be 0.736 μg/m^3^. At low concentration, the standard deviation of the PTI signal and the detection limit was 2.13 μV and 0.426 μg/m^3^, respectively. Figure 9b shows that the correlation was improved compared to Figure 8b. The caveat, however, is that the correlation could be negative for the data between 5 and 10 μV in Figure 9b. Figure 8b and Figure 9b show that the sensitivity was nominally 0.16–0.19 (μg/m^3^)/μV for high and low concentration cases. This value is lower than the sensitivity for spark-generated BC in the laboratory, which may have been caused by the coherence noise of optics (windows, lenses, etc.) or by aerosols deposited onto the surface of optics. Window optics are more easily exposed to various aerosols in ambient experiments than in laboratory experiments. Longer measurement time and uncontrollable humidity in ambient measurements may create coherent window noise.

### 3.6. Consideration of Factors Limiting Reliable Measurements

The sensitivity of the present PTI system may be limited by the degree of incoherence where the interference signal is less effectively produced. The interference depends on the degree of retardation controlled by the liquid crystal variable retarder. A failure of controlling the retardation may cause incoherence, resulting in low sensitivity. Detection sensitivity may be degraded by the combination of various noise sources because noise is enhanced to the extent that the noise level overwhelmed the PTI signal. Noise from the pump laser, mechanical noise inducing beam misalignment, and window surface effects due to periodic heating of various optics are contributing to the system noise which scales with the PTI signal.

The pump beam created by the DPSS laser sometimes deviated from the TEM_00_ mode, incompletely heating the measurement volume. Thus, the heating along the axis in the measurement volume interrogated by the probe is probably somewhat less effective than it would otherwise be the case. At a TTL modulation frequency of 83 Hz, the modulation period is short compared to the thermal diffusion time across the measurement volume (~1 mm) in air, where the thermal diffusion time of air is easily calculated to be ~50 ms from the air thermal diffusivity of 2.18 × 10^−5^ m^2^/s at 1 atm and 298 K. The lower sensitivity observed was mainly attributed to coherent window noise induced by absorbing aerosols deposited onto windows. This limitation is attributed to irregular and nonuniform heating of the now absorbing optics and the adjacent air layers by the non-TEM_00_ excitation beam. Again, careful design of a stable beam splitter and purging of windows with filtered air and better selection of pump laser should further enhance the capability of detecting BC using the PTI.

## 4. Conclusions

A polarization-based folded Jamin interferometer system was developed for the first time to characterize light-absorbing aerosols. Data have been obtained from polarization-based photothermal interferometry (PTI) of the black carbon (BC) particles generated in a commercial spark discharger. The sensitivity of the current PTI was estimated using the size-selected, 60-nm diameter, spark-generated BC particles. The signal obtained using our current PTI was compared with the eBC measured with a filter-based instrument, MAAP. On a high concentration day, the PTI signal showed a similar trend to the eBC concentration. On a low concentration day, however, MAAP overestimated the eBC concentration probably due to the high humidity caused by rainfall. Though the current PTI sensitivity is sufficient for the detection of ambient BC in urban areas, better sensitivity is required for the use of PTI in more pristine areas. The quadrature condition for optimum PTI operation is very sensitive to the input voltage of the variable retarder controller. Thus, a sensitive feed-back circuit is likely to improve the detection limit. The beam-dividing characteristics of the polarized beam splitter are important to set the quadrature conditions. Several issues regarding signal deterioration included the baseline increase, the stability of the pump laser, the stability of the liquid crystal retarder controller, and other more minor factors. Overcoming these issues will increase the sensitivity of PTI. Our results will help promote research on the monitoring of light-absorbing aerosols in the atmosphere.

## Figures and Tables

**Table 1:** Number concentration, average PTI signal, effective density estimated using data from [26], estimated mass concentration, sensitivity, and detection limit for 60-nm diameter spark-discharge generated black carbon.

Case	A		B		C	
Number concentration [#/cc]	2.93(±0.44) × 10^5^		6.77(±0.43) × 10^4^		6.44(±0.29) × 10^5^	
PTI signal [μV]	12.3 ± 2.85		3.55 ± 1.67		28.9 ± 1.96	
Effective density [g/cc]	0.403	0.428	0.403	0.428	0.403	0.428
Mass concentration [μg/m^3^]	13.4	14.2	3.09	3.28	2.94	3.12
Sensitivity^*^ [(μg/m^3^)/(μV)]	1.09	1.16	0.87	0.92	1.02	1.08
DL^**^ [μg/m^3^]	3.10	3.29	1.45	1.54	1.99	2.12

* Sensitivity was calculated as the ratio of the estimated mass concentration to the average PTI signal; ** DL (Detection limit) was calculated by multiplying the standard deviation of PTI signal by the mass concentration per unit PTI signal
